# Kinetic studies of complex biomedical process by magnetic resonance: Cuban experiences

**DOI:** 10.1007/s00723-018-0985-2

**Published:** 2018-03-26

**Authors:** Carlos Cabal Mirabal, Adolfo Fernández García, Manuel Lores Guevara, Evelio González, Leonardo Oramas Díaz

**Affiliations:** 1 Medical Biophysics Center, University of Oriente, Santiago de Cuba, Cuba; 2 Center for Genetic Engineering and Biotechnology, Havana, Cuba

## Abstract

The potentials of the magnetic resonance (MR) methods in the research of biomedical systems have been demonstrated during the 70 years of its existence. It is presented that the Cuban experience in quantitative magnetic resonance associated with molecular, preclinical and clinical studies of significant diseases and drugs development. MR “in vitro” and “in vivo” studies of sickle cell disease, the diabetic foot ulcer, the brain tumor response and the magnetic nano-particle pharmacokinetics, are presented as example. Furthermore, contributions and restrictions of magnetic resonance to diagnostic and optimization of therapeutic pathways are discussed in some concrete cases.

## Introduction

The potentials of the magnetic resonance (MR) methods in the research of biomedical system have been demonstrated during the 70 years of its existence, which it is celebrating during these years. It is presented that the Cuban experience in quantitative magnetic resonance associated with molecular, preclinical and clinical studies of significant diseases and drugs development.

MR “in vitro” and “in vivo” studies of sickle cell disease (SCD) [1–4], the diabetic foot ulcer (DFU) [5, 6], the brain tumor (BT) response [8] and the magnetic nano-particle pharmacokinetics (PK-MNP) [10] are presented as illustration. Furthermore, contributions and limitations of MR to diagnostic and selection of therapeutic pathways are discussed.

Common purposes of these investigations were to set up the necessary methodologies for the MR quantitative studies of complex pathological scenario.

## Materials and methods

### Study subjects

The present studies were entirely reviewed and approved by the ethics committee of the different medical institutions and of the research centers that are involved in, according to the ethical principles of the Word Medical Association (Declaration of Helsinki). The written informed consent of the healthy volunteers and the patient was obtained before inclusion in all cases. The studies were carried out following the ethical norms for the use of the laboratory animals; the Guides of Laboratory Good Practices and the Standard Operation Procedures.

The detailed description of the “Materials and methods” of MR “in vitro” studies of SCD is presented in Ref. [1–4]. Likewise, for the DFU, BT responses and PK-MNP have been published in Ref. [5, 6, 8, 10], respectively.

The main common idea of all these works is to optimize the experiments keeping, as constant as possible, all the conditions including the position of the biological sample. In each case, well-characterized external and internal markers, as references, have been establishing to have a control of the subject’s position and biological changes during the kinetic studies.

### SCD studies

In vitro magnetic relaxation studies were performed with a Cuban relaxometer. ^1^H *T*1 and *T*2 at 4 MHz were determinate using inversion recovery (IR), spin echo (SE) and Carr–Purcell–Meiboon–Gil (CPMG) pulse sequences with an error less than 5%. A Varian E-109 Century (X band) spectrometer was employed to determine the saturation transfer spectra of the hemoglobin (Hb) bound radical in HbA and HbS samples as is described in Ref. [3]. ^1^H relaxation and ESR measurements were performed during 8 h of the spontaneous deoxygenation of blood sample of more than 80 patients at 36 °C [1–4].

### DFU and BTR studies

MRI and MRS “in vivo” studies were performed with a 1.5 T Symphony Master Class system (Siemens). Before each study, the pulse sequences were optimized by means of phantom having *T*1 and *T*2 close to biological subject characteristics, as well as, with the contribution of healthy volunteers [5, 7, 8, 10].

A MR study of ten DFU patients were done before and during the intralesional epidermal growth factor (EGF) treatment until the lesion was totally closed [7].

A special device was created to be placed inside the head radiofrequency (RF) coil to guarantee and control the reproducibility of the feet positioning. It was demonstrated that the RF coil parameters (Q and B1 homogeneity) did not change [5, 6].

In the device, both feet are fix at the same time allowing simultaneously MRI images without changes in the experimental conditions. Each foot serves as a reference for the other. The device provides two affixed sets of external markers. These markers are connected with internal anatomic markers as a foot position Ref. [5, 6]. As well the device allows controlling the different anatomical foot structure position related to the cited external maker. In Ref. [5, 6] is discussed in more details the relationship between the internal and external marker for control of the foot position during the kinetic MRI studies.

Fourteen pediatric patients treated with monoclonal antibody nimotuzumab [9] were evaluated by MRI and MRS for more than 2 years. Each patient was their own control [8].

### PK-MNP chemically modified with polyethylene glycol (PEG)

Measuring the signal noise ratio (SNR), contrast noise ratio (CNR) and non-images uniformity (NUI) in 1.5 T MRI clinical machine was selected the best RF coil. It was achieved using a phantom having the same dimension and similar *T*1 and *T*2 characteristics of rats. Between the head and flexible coils, the flexible one was better by more than 25%.

MNP solutions relaxivities (*r*_1_ and *r*_2_) was determinate “in vitro” and “in vivo” by MR relaxation and MRI [10].

Before the MRI examination, all Wistar rats (*n* = 4) have been weighted to determinate the MNP and the anesthetic doses as described in Ref. [10]. The MNP-PEG-(NH2)2 doses used was 5 mg/kg (*n* = 2) and 2.5 mg/kg (*n* = 2) of animal weight. Before, all the MNP samples were homogenized by ultrasound for 2 min. The injection of the NP-PEG-(NH2)2 was through tail vein. Immediately after the anesthesia, the two rats were collocated and immobilized in a rubber special device enclosed in the RF coil. The rat’s couple in one RF coil permit to increase the SNR. At the same time, one rat was reference for the other, in exactly the same conditions during the experiment.

Each couple of rats have been MRI scanned simultaneously just before the injection of NP-PEG-(NH2)2. These images have been considerate as the *t* = 0 images. After the first and single injection, the rat’s liver MRI imaging were scanned periodically until 528 h (22 days).

## Results and discussion

### MR relaxation and electron spin resonance studies of SCD

SCD is the first molecular disease described. Due to the polymerization of HbS, the erythrocyte is deformed, the permeability and elasticity of the membrane change, hemolysis is caused and rheology is modified, leading to painful vaso-occlusive crises [1, 4].

**Fig. 1:**
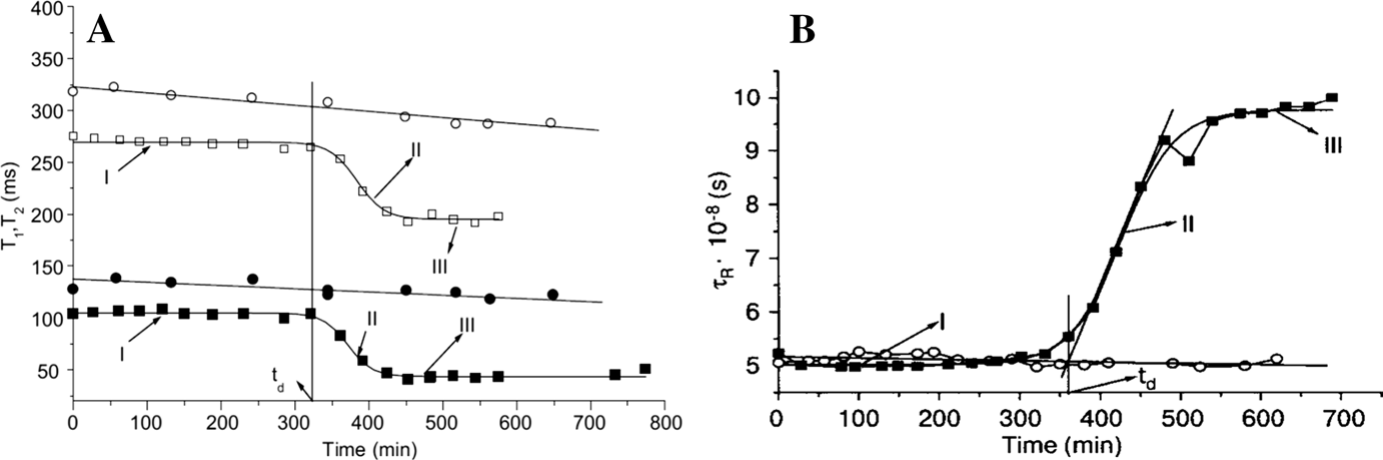
**a** Typical *T*1and *T*2 temporal behaviors in HbA and HbS samples (at intracellular concentration and 36 °C). The continuous lines represent the linear and sigmoidal fittings corresponding to the HbA and HbS samples, respectively. In HbS, stage I corresponds with the nucleation, where most molecular aggregation are reversible; stage II corresponds with the irreversible development of polymerization, and the stage III, with the formation of micro-domains [2]. **b** Typical behavior of $\tau_{R }$ in samples studied by ESR [3].The solid lines represent the sigmoidal and linear fitting that characterize the result obtained in HbS and HbA, respectively. Filled square: *T*2 HbS, open square: *T*1 HbS, open circle: *T*1 HbA, filled circle: *T*2 HbA [2, 3]

Typical *T*1 and *T*2 temporal behaviors in HbA and HbS samples are shown in Fig. 1a [2, 3]. The kinetic of deoxyHbS polymerization is characterized by three stages: initial (I), nucleation (II) and termination (III) (1–4). The initial phase is delimited by the delay time (*t*_d_) [1–4]. After, the *t*_d_ takes place an autocatalytic HbS polymerization. Once nucleation concludes, ^1^H relaxation tends to stabilize (III). The different MR behavior in HbA and HbS solutions could be associated with: (1) the molecular mobility changes. (2) Hb’s magnetism variations (oxyHb: diamagnetic and deoxyHbS: paramagnetic) and (3) the appearance of micro in homogeneities at the end of the polymerization process [2, 3]. However, the relative constant of the *T*1 and *T*2 for HbA samples excludes the possibility of HbS magnetism variations influence of the magnetic properties of the Hb complexes.

The different possible relaxation mechanism was discussed in Ref. [2] concluding that probably the dipole–dipole interactions is the most important. If it is assumed that the dipole–dipole interaction is the main mechanism then from the *T*1, *T*2 and *T*1/*T*2 ratio values, in the regions I–III, can make an assessment and conclude that $\tau_{\text{C}} \approx 4 \times 10^{ - 8} {\text{s}}$.

A more precise quantitative evaluation of $\tau_{\text{C}}$ variation was done by ESR. Table 1 and Fig. 1b present the values and temporal behavior of $\tau_{\text{R}}$ and $\eta_{\mu }$ determinate by ESR [3]. It is a good agreement between the ^1^H MR relaxation and ESR by the spin labeling method data.

**Table 1:** Rotation correlation time and micro-viscosity determined by ESR [3]

Parameter	Región I (HbS)	Región II (HbS)	Región III (HbS)	HBA
$\tau_{\text{R}}$ (× 10^−8^)	5 ± 0.11	5 ± 0.11 ≤ $\tau_{\text{R}}$ ≤ 9.8 ± 0.22	9.8 ± 0.22	5.8 ± 0.13
*ƞ* (mPas)	2.06 ± 0.1	2.06 ± 0.1 ≤ *ƞ* ≤ 3.79 ± 0.19	3.79 ± 0.19	2.06 ± 0.1

The strong existing correlation between the MR parameters (*T*1, *T*2,$\tau_{\text{R}}$, $\eta_{\mu }$) with the $t_{\text{d}}$ has allowed us to stablish a new diagnostic method for the differentiation between the crises and steady stages of the patients [4].

**Fig. 2:**
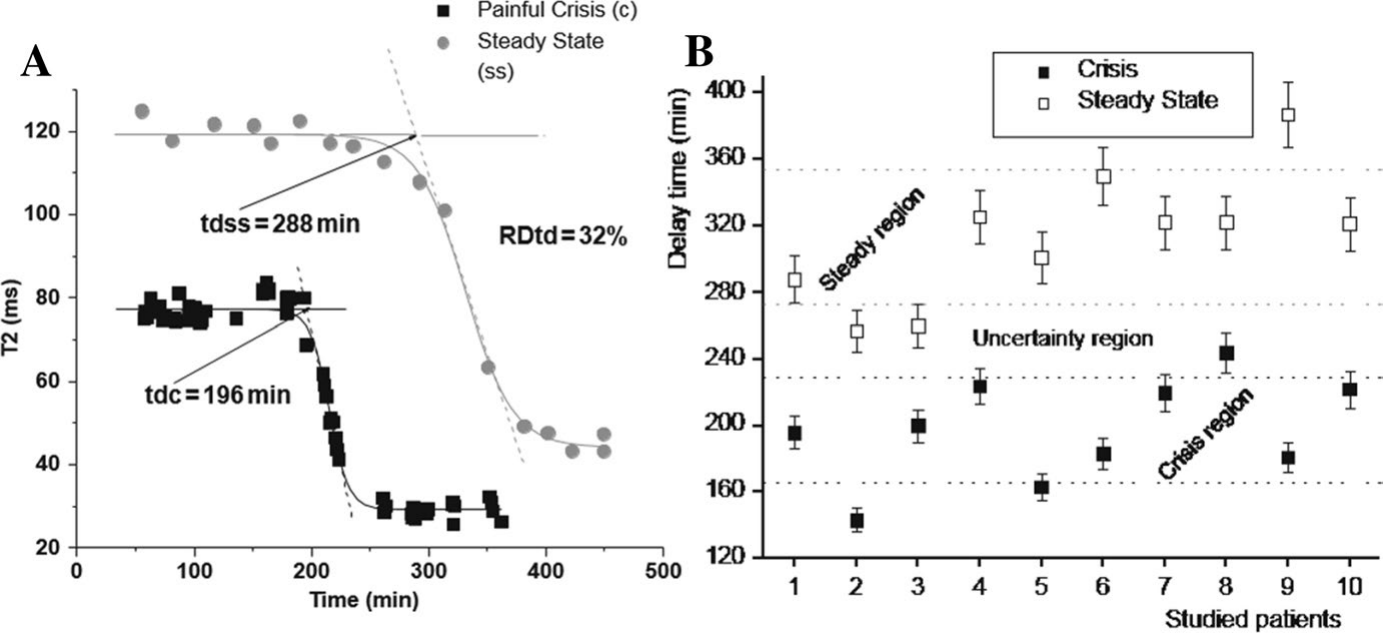
**a** Typical temporal behavior and *t*_d_ values related the steady stage and in painful crisis clinical conditions, for the same pediatric patient. The black box curves correspond to the crisis. The relative differentiation between both *t*_d_ values is 32% for this case [4]. **b** Delay time values corresponding to the steady and painful crisis clinical conditions stages for ten pediatric sickle cell patients. The gray horizontal lines represent the *t*_d_ limits corresponding to the steady and crisis regions. The uncertainty region includes the 15% of the measured values [4]

A decrease of *t*_d_ (32%) can be observed during painful crisis conditions in the same sickle cell patients (see Fig. 2a). As summarized in Fig. 2b, a similar result was obtained for all patients studied under painful crisis conditions, in which the mean value of the relative differentiation of *t*_d_ with respect to steady state condition is 36 ± 10%. It means that it can be established a differentiation between both clinical conditions when you compare the *t*_d_ value of one patient during crisis with his own *t*_d_ value during steady state. As it is concluded in Ref. [4] ^1^H relaxation method can be used to differentiate steady state and crisis clinical conditions.

Furthermore, this MR procedure made possible the discovery of the anti-sickling action of 4-hydroxy-3-methoxybenzaldehyde (Vanillin, a nontoxic compound) as a therapeutic drug, as it was established in Ref. [1, 4]. The anti-sickling action on polymerization of vanillin was demonstrated “in vivo” in a double blind, placebo-controlled clinical therapeutic trial performed in 30 patients (11 belonging to the control group), through the increment of the *t*_d_ on average 1.6 times indicating a decrease in the effectiveness of the polymerization process [1, 4].

### MRI/MRS quantitative evaluation of DFU response under treatment in clinical studies

The changes of DFU lesion sizes (area and volume), edema (volume), as well as apparent diffusion coefficient (ADC), and metabolites spectra, as a function of the EGF treatment time, were reported during a clinical trial phase IV [5].

**Fig. 3:**
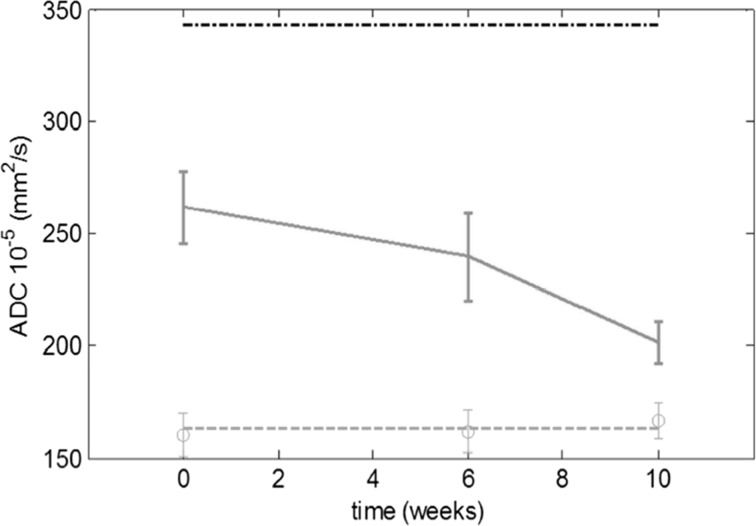
Calculated ADC from DWI, as a function of the treatment time (in weeks), for one patient. The ADC of the healthy foot is shown in red. The blue line represents the ADC of the DFU and the black line denotes the free water ADC [5]

As example in the Fig. 3, the ADC data as a function of the treatment time is presented for one of the patient. The described device fixes both feet at the same time allowing MRI images simultaneously. Then, each foot can be a reference for the other [5, 6]. In Fig. 3 are shown the three ADC time-dependent curves corresponding to: healthy foot (in red), DFU (in blue), and the free water ADC (in the top, in black), as control. These plots demonstrate that DFU ADC curves show a trend toward the healthy foot values during the treatment time.

If the measurements are performed under identical conditions (foot position, pulse sequence, slice orientation, etc.), we could hypothesize that ADC-relative changes are connected to the tissue texture differences and others physio-pathological features; regardless of ADC complex dependence on tissue characteristics. This was the first report of ADC and edema evolution of the DFU under treatment. The ADC data are in agreement with the edema that reported each micro-region or voxel in the foot. Whichever, this procedure is useful for evolution studies of other pedal and tissue disorders [5].

All MRI/MRS data obtained are in accordance with clinical observation. Moreover, the obtained quantitative information permits to understand several healing mechanism at tissue level and to optimize the treatment procedure.

### MRI/MRS quantitative evaluation of BT response under treatment in clinical studies

The characterization of the evolution of the tumor lesion was made from the following magnitudes and parameters obtained from MRI:

1. Volume of the lesion visualized in the *T*1 (*V*_*T*1_), *T*2 (*V*_*T*2_) and FLAIR (*V*_FLAIR_) images. A more accurate evolutionary study is possible using these different volumes, as they are associated with different tumor cell densities and textures. Using these different volumes, a more accurate evolutionary study is possible as a consequence that they associated with different tumor cell densities and textures. The following volume ratios were calculated: *V*_*T*2_/*V*_*T*1_, *V*_FLAIR_/*V*_T1_, *V*_*T*2_/*V*_FLAIR_.

**Fig. 4:**
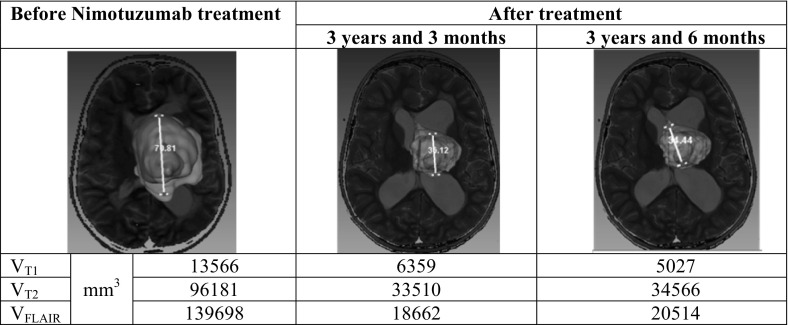
Illustration of a calculated tri-dimensional representation of images of patient no. 12 with a brain stem tumor. The lesion is labelled in *T*1_Gd_ (red), *T*2 (magenta) and FLAIR (purple) [8]

An example of lesion volume evaluation (*V*_T1_, *V*_T2_ and *V*_FLAIR_) in patient 12 is given in Fig. 4.

These different volumes correspond to diverse molecular mobility and cell densities in the tumor region. Evidently, there are complex correlations between the relaxation times, the different mean spectral density function values and the physio-pathological characteristics of the tumor response. Nevertheless, the comparison between lesion volumes obtained from different image contrast contains more information about the lesion state, beyond the simple volume acquired from one image type with and without CA.

The real quantitative separation of the different molecular and cellular mobilities associated with the different pathological tissue regions is one of the unsolved problems regarding these complex pathologies. Many efforts are been made to overcome this essential problem for MRI diagnostic. It is possible to attenuate, or in some cases, to avoid this limitations having robust protocol, decreasing the image time, using external and internal markers and by means of multi-parametric images. Nowadays, a real *T*1, *T*2 “in vivo” map is a great challenge to the quantitative MRI.

2. In the previous mentioned sense, the ADC maps are important information to the understanding of the relaxation and molecular mechanism connected to each different tumor regions. Quantitative ADC maps, related to the water mobility, for the lesion and for its surroundings were obtained for the patients under study, before and during the treatment [8].
3. Amplitude ratios of *N*-acetyl aspartate (NAA), choline (Cho) and creatine metabolite peaks. Lesion and healthy areas located in the contra-lateral hemisphere were studied. The NAA/Cr, Cho/NAA and Cho/NAA ratios were retrospectively evaluated for each patient, and used as one of the evaluating criteria [8].

The present MRI/MRS results are in good agreement with the clinical evaluation and confirm once again the MR possibilities when a standardized robust protocol is used [8]. The MRI/MRS data gave distinctive complementary information related to the BT response during long treatment time to delimit the treatment conduct and contribute in the understanding of bio-physiological process associated with the anti-tumor mechanism of the drugs.

### MNP. Pharmacokinetics (PK) by MR relaxation and MRI

Nowadays, MNPs are applied for diagnosis, drug delivery and thermotherapy agents amongst others. MRI is a complementary technique to follow PK of MNP [10].

**Fig. 5:**
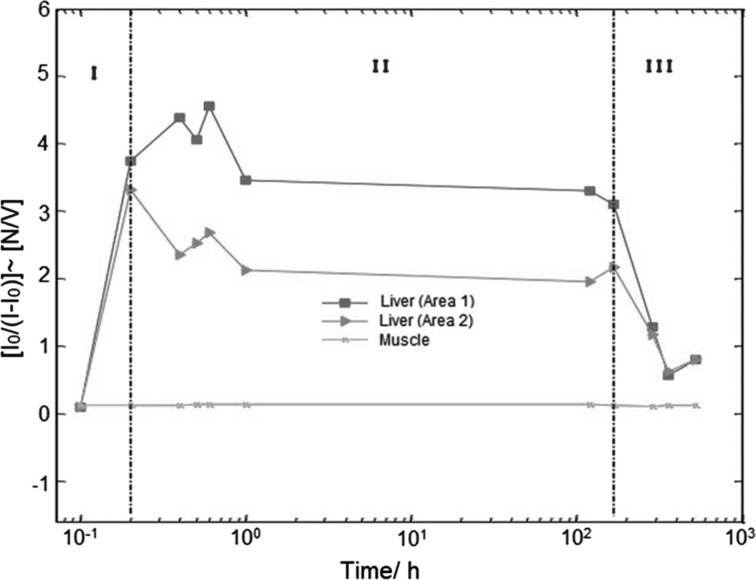
Dependence of the *I*_0_/(*I*_*i*_ − *I*_0_) ratio (proportional to the MNP concentration) as a function of time after MNP injection. Two different areas of the liver were measured (red and blue lines) and, as a control, a muscle area was taken (line pink line) [10]

In Fig. 5 is represented the dependence of the ratio (*I*_*i*_ − *I*_0_)/*I*_0_, proportional to MNP concentration. The measured image intensities *I*_*i*_ were normalized to the background *I*_0_ and the ratio (*I*_*i*_ − *I*_0_)/*I*_0_ was calculated as a time function after MNP injection. Two different areas of the liver were evaluated (red and blue lines) and one muscle area (pink line) was taken as control. The graphics have three regions with different slopes. The first one, which is associated with the entrance of the MNP in the liver: absorption period. The second one can be attributed to the liver stationary state: residence. Finally, the last region is connected to the MNP excretion. From these measurements, it was possible to determine the principal MNP PK parameters as reported in Ref. [10].

A new metabolic route of MNP “in vivo” bio-distribution was described in base of this data. Nonetheless, the MNP MR “in vivo” studies are limited as consequence of the strength “negative contrast” introduced by MNP in *T*2 relaxation mechanism. It is necessary to make some complementary effort to overcome this restriction.

## Conclusion

“In vitro” and “in vivo” MR data gave unique complementary information related to the kinetic studies of complex biomedical processes. MR has contributed to the discovery of new pathways and drugs for treatment and to introduce modification in them. Furthermore, MR provides valuable information on bio-processes that occur over long periods of time, contributing to the understanding of the physiological processes and delimiting the treatment conduct.

Nonetheless, the real quantitative separation of the different mobility’s (molecular and cellular), the metabolic activity, associated with the different pathological tissues regions still be an unsolved problem related to complex pathologies. This essential and complex MRI problem need many effort to be overcome. Nowadays, to obtain a real quantitative *T*1, *T*2, Diffusion and metabolic maps “in vivo”, in real time, is still a great challenge to the MR.

Nevertheless, it is possible to attenuate, or some cases to avoid these restrictions by having robust and standard protocols, decreasing the image time, by means of external and internal markers, employing adequate and well-characterized phantoms and via the multi-parametric images among other procedures. In addition, adequate preparation for the technicians and medical experts working in MR is a success factor for achieving these proposes.
